# Curcumin Combined with FOLFOX Chemotherapy Is Safe and Tolerable in Patients with Metastatic Colorectal Cancer in a Randomized Phase IIa Trial

**DOI:** 10.1093/jn/nxz029

**Published:** 2019-05-27

**Authors:** Lynne M Howells, Chinenye O O Iwuji, Glen R B Irving, Shaun Barber, Harriet Walter, Zahirah Sidat, Nicola Griffin-Teall, Rajinder Singh, Nalini Foreman, Samita R Patel, Bruno Morgan, William P Steward, Andreas Gescher, Anne L Thomas, Karen Brown

**Affiliations:** 1 Leicester Cancer Research Centre, University of Leicester, Leicester, United Kingdom; 2 Leicester Clinical Trials Unit, University of Leicester, Leicester, United Kingdom; 3 Hope Clinical Trials Facility, Leicester Royal Infirmary, Leicester, United Kingdom

**Keywords:** metastatic colorectal cancer, FOLFOX chemotherapy, curcumin, curcuminoids, randomized controlled trial

## Abstract

**Background:**

Curcumin is the main active ingredient of the spice turmeric, investigated extensively for putative anticancer properties.

**Objectives:**

This phase IIa open-labelled randomized controlled trial aimed to assess safety, efficacy, quality of life, neurotoxicity, curcuminoids, and C-X-C-motif chemokine ligand 1 (CXCL1) in patients receiving folinic acid/5-fluorouracil/oxaliplatin chemotherapy (FOLFOX) compared with FOLFOX + 2 g oral curcumin/d (CUFOX).

**Methods:**

Twenty-eight patients aged >18 y with a histological diagnosis of metastatic colorectal cancer were randomly assigned (1:2) to receive either FOLFOX or CUFOX. Safety was assessed by Common Toxicity Criteria-Adverse Event reporting, and efficacy via progression-free survival (PFS) and overall survival (OS). Quality of life and neurotoxicity were assessed using questionnaires (European Organization for Research and Treatment of Cancer Quality of Life Questionnaire-C30 and Functional Assessment of Cancer Treatment-Gynecologic Oncology Group-Neurotoxicity). Plasma curcuminoids were determined with liquid chromatography (LC) electrospray ionization tandem mass spectrometry and CXCL1 by ELISA.

**Results:**

Addition of daily oral curcumin to FOLFOX chemotherapy was safe and tolerable (primary outcome). Similar adverse event profiles were observed for both arms. In the intention-to-treat population, the HR for PFS was 0.57 (95% CI: 0.24, 1.36; *P* = 0.2) (median of 171 and 291 d for FOLFOX and CUFOX, respectively) and for OS was 0.34 (95% CI: 0.14, 0.82; *P* = 0.02) (median of 200 and 502 d for FOLFOX and CUFOX, respectively). There was no significant difference between arms for quality of life (*P* = 0.248) or neurotoxicity (*P* = 0.223). Curcumin glucuronide was detectable at concentrations >1.00 pmol/mL in 15 of 18 patients receiving CUFOX. Curcumin did not significantly alter CXCL1 over time (*P* = 0.712).

**Conclusion:**

Curcumin is a safe and tolerable adjunct to FOLFOX chemotherapy in patients with metastatic colorectal cancer. This trial was registered at clinicaltrials.gov as NCT01490996 and at www.clinicaltrialsregister.eu as EudraCT 2011-002289-19.

## Introduction

Colorectal cancer remains a significant contributor to morbidity and mortality in the UK, with ≤20% of patients presenting with metastatic disease (1). Surgical resection in those individuals with anatomically isolated metastases can significantly improve survival outcomes, but is feasible in <30% of patients (2). Combination 5-fluorouracil (5-FU) chemotherapy with oxaliplatin or irinotecan and addition of anti-epidermal growth factor receptor agents in patients with rat sarcoma wild-type disease remains the standard of care, extending median survival to almost 30 mo.

Although targeted therapies have the facility to provide clear benefit in specific patient cohorts, there is a need to investigate alternative strategies using low-toxicity multitarget agents. Such agents may be advantageous for use in frailer populations and have fewer economic limitations in increasingly cost:benefit ratio–driven healthcare settings. Repurposed drugs and diet-derived agents with well-characterized pharmacokinetic and toxicological profiles potentially offer improved quality of life and clinical benefit, yet clinical data are sparse. One such diet-derived agent is curcumin, derived from the spice turmeric.

Curcumin pharmacokinetics and safety have been extensively profiled in healthy volunteer and clinical populations. Window-of-opportunity studies in colorectal cancer patients have established both safety and tolerance in patients awaiting surgical resection of their cancer (3, 4), and that pharmacologically active concentrations of curcumin are detectable in bowel mucosa after oral administration (5). Furthermore, potential for beneficial effect has been observed in patients with aberrant crypt foci, with significant reduction in aberrant crypt foci numbers after 4 g curcumin/d for ≤35 d (6). Many mechanism-driven hypotheses have been established to explain curcumin's efficacy in vitro, in vivo, and in patient-derived ex vivo models. Putative anticancer efficacy of curcumin is thought to be driven by numerous multitargeting mechanisms, including the ability to decrease inflammatory mediators, cellular proliferation, angiogenesis, and metastatic spread, and to increase cell cycle arrest and apoptosis (7–10). Potential for utility of curcumin as a low-toxicity adjunct to chemotherapy has also been investigated in preclinical models. We previously established that curcumin significantly increased apoptosis and decreased proliferation in colorectal cancer cell lines when combined with oxaliplatin (11), which was recapitulated in vivo using the HCT116 xenograft mouse model (12). Ex vivo tissue explants from patients with colorectal liver metastases showed greater sensitivity to the combination of curcumin with the chemotherapy drugs oxaliplatin and 5-FU than to chemotherapy alone, likely by targeting the cancer stem cell population (7). Curcumin-mediated response in explant models was also found to be associated with high tissue expression of the chemokine C-X-C motif chemokine ligand 1 (CXCL1) (13).

Potential to translate preclinical anticancer effects into clinical benefit has been investigated for a number of cancer sites by combining curcumin with standard-of-care chemotherapy, yet, to date, no randomized controlled trials have been undertaken. In addition, with patients often turning to self-administration of dietary supplements (14, 15), it is important to establish whether drug–supplement interactions may compromise chemotherapeutic safety or efficacy.

More recently, we showed that addition of daily oral curcumin (0.5–2.0 g) to folinic acid/5-fluorouracil/oxaliplatin (FOLFOX) chemotherapy in patients with colorectal liver metastases was safe and tolerable in a phase I dose escalation study (7). We present here the results of a phase IIa open-labelled, 2-armed, randomized controlled trial, comparing efficacy of first-line FOLFOX chemotherapy alone with that of FOLFOX plus curcumin (CUFOX), in patients with colorectal metastases. The primary outcome of this study was to further evaluate safety and tolerability of this combination, in addition to determining the potential for any clinical benefit (secondary outcome).

## Methods

The full trial protocol in accordance with the Standard Protocol Items: Recommendations for Interventional Trials (SPIRIT) and Consolidated Standards of Reporting Trials (CONSORT) statements (16, 17) has been previously reported (18), with ethical approval for this study granted by the East Midlands (Derby 1) regional ethics committee (11/EM/0263). This phase IIa open-labelled randomized controlled trial was conducted in accordance with the International Conference on Harmonization - Good Clinical Practices (ICH-GCP) and the Declaration of Helsinki. In brief, patients with a histological diagnosis of metastatic colorectal cancer awaiting first-line chemotherapy were recruited at the University Hospitals of Leicester Oncology Department and randomly assigned (at a ratio of 1:2) to receive standard-of-care chemotherapy (at that time) (FOLFOX ± bevacizumab) or FOLFOX ± bevacizumab plus 2 g oral Curcumin C3 Complex/d (Sabinsa Corp—containing ∼80% curcumin and 20% demethoxycurcumin and bisdemethoxycurcumin, encapsulated by Nova Laboratories) (CUFOX). Chemotherapy was given once every 2 wk for ≤12 cycles or until patient progression, unacceptable toxicity, death, or withdrawal.

After trial commencement, there were 2 protocol changes relating to inclusion criteria: enabling recruitment of patients with known peptic ulcer disease; and not restricting to patients with proven liver disease only. At the time of study inception, exclusion of patients with peptic ulcer disease was taken as a precaution owing to the potential for curcumin to exacerbate gastrointestinal symptoms in the presence of chemotherapy. Emerging evidence suggested that curcumin was unlikely to contribute to peptic ulcer disease (19). The lung is the second most common metastatic site in colorectal cancer, so the inclusion criteria were expanded to reflect this.

Recruitment ceased at 28 of the 33 participants specified in the original protocol. During the trial, new clinical recommendations gave patients alternate options to FOLFOX chemotherapy, meaning that the ability to complete recruitment was compromised. The investigators took the decision to discontinue the study because prolonged recruitment times would have resulted in curcumin capsules being out of date.

### Curcuminoid analysis

Quantitative analysis of curcuminoid concentrations in plasma was performed using our established LC electrospray ionization MS/MS assay, which detects both parent curcumin and its major metabolites (20). Data were acquired using MassLynx software (Waters, UK) version 4.0 and concentrations of curcumin and metabolites were determined from individual calibration lines constructed from use of curcumin, curcumin glucuronide, and curcumin sulfate standards.

### CXCL1 analysis

Plasma CXCL1 [growth-regulated oncogene (GRO)-α] concentrations were analyzed using the Quantikine ELISA human CXCL1/GRO-α immunoassay (R&D Systems) as per the manufacturer's instructions. Assay diluent (RD1U) was added to each well, followed by 200 μL of standard, control, or plasma samples before incubating at room temperature for 2 h. Wells were aspirated, washed thrice before addition of human GRO-α conjugate, and incubated for 2 h at 2–4°C. The aspiration and washing steps were repeated, 200 μL of substrate solution was added, and the ELISA plate was incubated for 30 mins at room temperature in the dark. After addition of 50 μL stop solution, optical density was measured at 450 nm with wavelength correction at 540 nm, using a Fluostar Optima plate reader (BMG Biotech).

### Trial statistics

The statistical plan was drawn up by the Leicester Clinical Trials Unit following guidelines published by the American Statistical Association and the Royal Statistical Society for statistical practice (21, 22). Demographic variables are summarized by treatment arm and overall as mean (range) for continuous variables and *n* (%) for categorical variables. Safety outcomes including incidence rate of adverse events (AEs) were compared by treatment arm using a Wilcoxon Mann-Whitney test in the safety population. The primary analyses of efficacy outcomes were carried out in the intention-to-treat (ITT) population; these analyses were repeated in the per-protocol (PP) population. Ordinal and continuous efficacy outcomes for Quality of Life Questionnaire-C30 scores, neurotoxicity scores, and curcuminoid and CXCL1 concentrations across time points were also compared between treatment arms using Wilcoxon Mann-Whitney tests because they did not follow a normal distribution in each case. Overall survival (OS) and progression-free survival (PFS) were compared between treatment arms using log-rank tests. Progression was defined as death, a 30% increase in the target lesion, or the appearance of new nontarget lesions. Objective response was compared between treatment arms using a chi-squared test. Objective response was defined as ≥30% decrease in the sum of the diameters of target lesions at the scan of concern, taking as reference the baseline sum diameters with no appearance of new lesions and no progression of nontarget lesions at that scan or any scan between that scan and baseline. Pearson's correlation coefficients were calculated for pairs of continuous variables hypothesized to be correlated. Results were considered to be significant where *P* ≤ 0.05.

## Results

**FIGURE 1:**
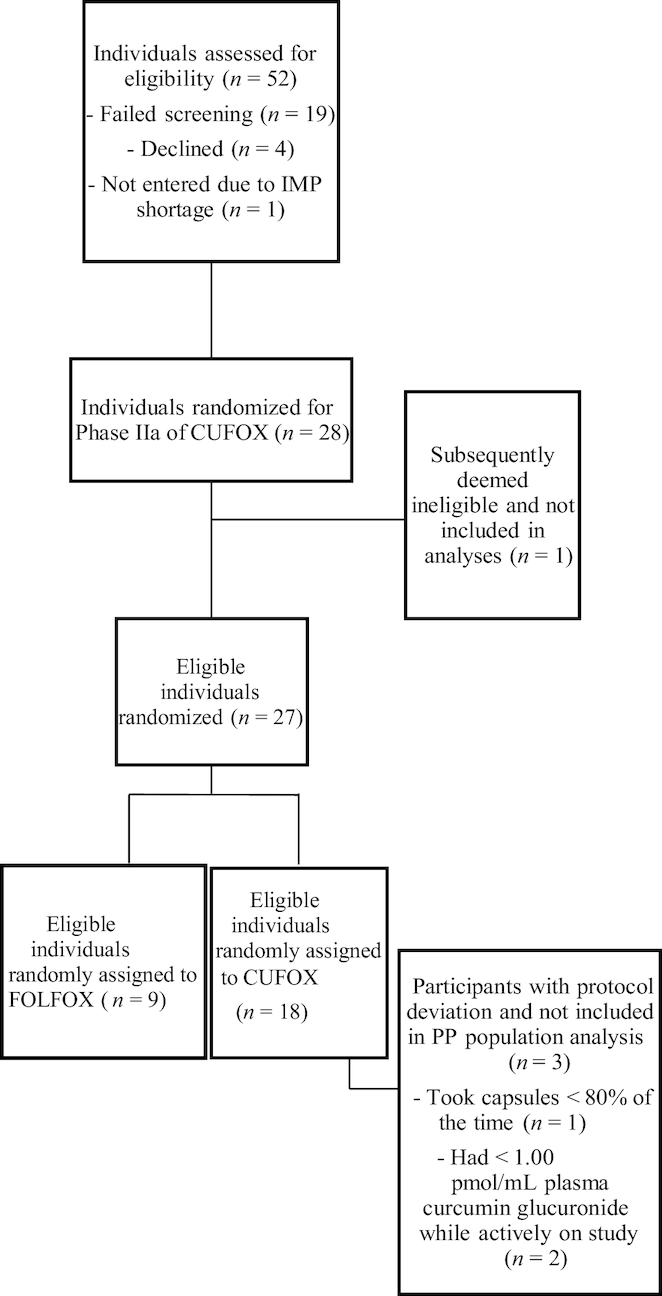
Consolidated Standards of Reporting Trials (CONSORT) diagram showing study participation, random assignment, and PP completion for the CUFOX study. Values represent frequency of events. CUFOX, folinic acid/5-fluorouracil/oxaliplatin + 2 g oral curcumin/d; FOLFOX, folinic acid/5-fluorouracil/oxaliplatin; IMP, investigational medicinal product; PP, per protocol.

From April, 2013 to May, 2016, 28 patients with stage IV disease were recruited and randomly assigned to take part in this study, of whom 9 (as one patient was subsequently deemed ineligible) were randomly assigned to FOLFOX ± bevacizumab, and 18 to FOLFOX ± bevacizumab plus curcumin (CUFOX) (**Figure 1**). Primary clinical data for the ITT population are summarized in **Table 1**. Mean age and performance status were similar in both arms. The main metastatic sites in both groups were liver and lung, with ≥2 metastatic sites observed in 77.8% of FOLFOX and 44.5% of CUFOX participants. Peritoneal disease was observed in CUFOX patients only.

**TABLE 1:** Demographics for patients with metastatic colorectal cancer receiving FOLFOX or CUFOX treatment^1^

	FOLFOX	CUFOX
Age, y	68.3 (56–78)	66.9 (53–77)
Resident in United Kingdom		
Whole of life	9 (100)	17 (94.4)
Part of life	0 (0)	1 (5.6)
White British	9 (100)	18 (100)
Performance status^2^		
0	6 (66.7)	11 (61.1)
1	3 (33.3)	7 (38.9)
Tumor staging^3^		
M1a	2 (22.2)	9 (50)
M1b	7 (77.8)	5 (27.8)
M1c	0 (0)	4 (22.2)
Metastatic sites		
Liver	9 (100)	17 (94.4)
Lung	7 (77.8)	6 (33.3)
Peritoneal	0 (0)	4 (22.2)
Adrenal	1 (11.1)	1 (5.6)
Portal hepatic nodes	1 (11.1)	0 (0)
Number of metastatic sites		
1	2 (22.2)	10 (55.6)
2	5 (55.6)	7 (38.9)
3	2 (22.2)	1 (5.6)
Additional bevacizumab	4 (44.4)	8 (44.4)

1 Values are mean (range) for age, and *n* (%) per arm for all other variables. The number of patients on FOLFOX and CUFOX in the intention-to-treat population was 9 and 18, respectively. CUFOX, folinic acid/5-fluorouracil/oxaliplatin + 2 g oral curcumin/d; FOLFOX, folinic acid/5-fluorouracil/oxaliplatin.

2 Eastern Cooperative Oncology Group definition: performance status of 0 = fully active, able to carry on all predisease performance without restriction; performance status of 1 = restricted in physically strenuous activity but ambulatory and able to carry out work of a light or sedentary nature, e.g., light house work, office work.

3 All participants were stage 4, but with varying metastatic (M) disease.

### Safety

Curcumin in combination with FOLFOX ± bevacizumab was generally well tolerated. FOLFOX participants received a median of 3 cycles of chemotherapy (mean: 5.1; range: 0–12) and CUFOX participants received a median of 12 cycles (mean: 9.4; range: 0–12). Three patients on curcumin received a 25% dose-reduction in 5-FU/oxaliplatin and 1 participant stopped curcumin early owing to nausea. A total of 103 AEs for FOLFOX and 282 for CUFOX were reported and those occurring in ≥2.5% of patients are shown in **Table 2**. Fatigue and peripheral neuropathy were the most common and were grade 1 or 2 in severity. The most common grade 3 or 4 toxicity was that of thromboembolic events, occurring in 3 patients receiving CUFOX. When expressed as “per cycle” events there was no significant difference between groups, with a mean of 0.7 serious AEs for FOLFOX compared with 0.3 serious AEs for CUFOX (*P* = 0.521), and 2.4 AEs for FOLFOX compared with 2.1 AEs for CUFOX (*P* = 0.142). AEs where causality was reported as possibly or probably related to curcumin were primarily gastrointestinal in nature, with the most common being diarrhea (**Table 3**). **Supplemental Table 1** shows the number of AEs reported by grade and arm. Global health scores did not show any significant difference between the CUFOX and FOLFOX arms (*P* = 0.248) (**Supplemental Figure 1**). A positive correlation between overall neurotoxicity scores and number of chemotherapy cycles (Pearson's correlation coefficient = 0.429, *P* = < 0.001) was observed, but no significant differences were observed in neurotoxicity scores between arms at cycles 6 (*P* = 0.223) or 12 (*P* = 0.204).

**TABLE 2:** Total number of AEs by arm and overall for patients with metastatic colorectal cancer receiving FOLFOX or CUFOX treatment^1^

	FOLFOX	CUFOX	Total
Constipation	4 (3.9)	7 (2.5)	11 (2.9)
Diarrhea	6 (5.8)	12 (4.3)	18 (4.7)
Dyspepsia	4 (3.9)	10 (3.6)	14 (3.6)
Fatigue	8 (7.8)	26 (9.2)	34 (8.8)
Nausea	5 (4.9)	8 (2.8)	13 (3.4)
Oral mucositis	5 (4.9)	10 (3.6)	15 (3.9)
Peripheral sensory neuropathy	6 (5.8)	25 (8.9)	31 (8.1)
Platelet count decreased	4 (3.9)	13 (4.6)	17 (4.4)

1 Values are *n* (%) of all AEs in each arm and overall for the intention-to-treat population, reported for those events that constituted ≥2.5% of all AEs. Number of patients on FOLFOX and CUFOX was 9 and 18, respectively. AE, adverse event; CUFOX, folinic acid/5-fluorouracil/oxaliplatin + 2 g oral curcumin/d; FOLFOX, folinic acid/5-fluorouracil/oxaliplatin.

**TABLE 3:** AEs possibly or probably attributable to curcumin by arm and overall for patients with metastatic colorectal cancer receiving FOLFOX or CUFOX treatment^1^

	FOLFOX	CUFOX	Total
Abdominal pain	0	2 (3.4)	2 (3.4)
Acute kidney injury	0	2 (3.4)	2 (3.4)
Anorexia	0	4 (6.8)	4 (6.8)
Bloating	0	2 (3.4)	2 (3.4)
Constipation	0	5 (8.5)	5 (8.5)
Diarrhea	0	10 (17.0)	10 (17.0)
Dry mouth	0	2 (3.4)	2 (3.4)
Dyspepsia	0	7 (11.9)	7 (11.9)
Flatulence	0	2 (3.4)	2 (3.4)
Nausea	0	8 (13.6)	8 (13.6)
Oral mucositis	0	6 (10.2)	6 (10.2)
Vomiting	0	5 (8.5)	5 (8.5)

1 Values represent *n* (%) of all AEs possibly or probably attributable to curcumin in each arm and overall for the intention-to-treat population, reported for those events that constituted ≥2.5% of all AEs. The number of patients on FOLFOX and CUFOX was 9 and 18, respectively. AE, adverse event; CUFOX, folinic acid/5-fluorouracil/oxaliplatin +2 g oral curcumin/d; FOLFOX, folinic acid/5-fluorouracil/oxaliplatin.

### Efficacy

Of the 27 patients in the study, 22 died during the follow-up period: 9 of 9 on FOLFOX and 13 of 18 on CUFOX. One patient from each arm did not commence chemotherapy owing to rapid disease progression. Four participants proceeded to surgical resection of liver metastases (2 on FOLFOX, 2 on CUFOX) between cycles 7 and 12 of chemotherapy.

**FIGURE 2:**
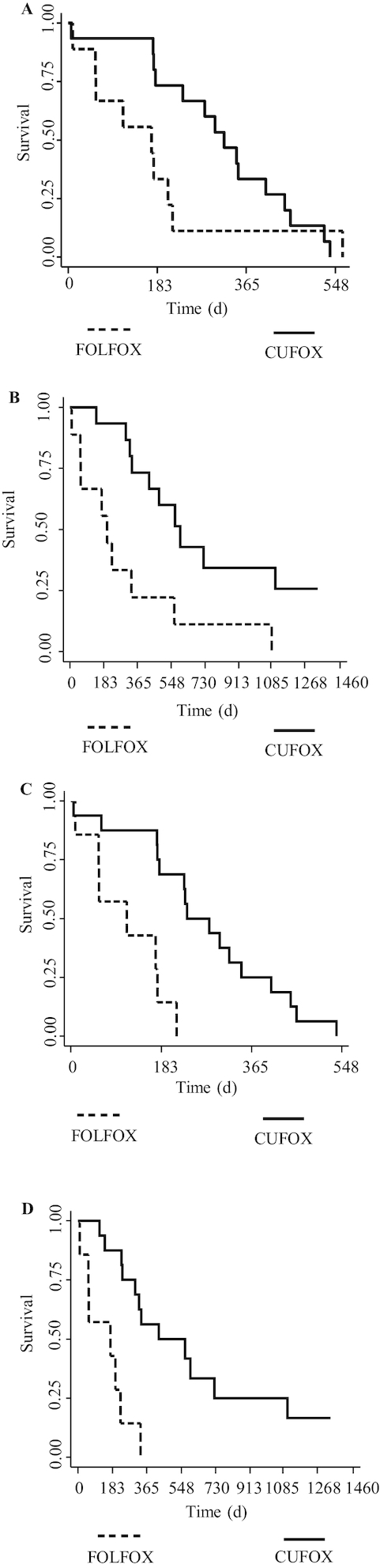
Efficacy outcomes for patients with metastatic colorectal cancer receiving either FOLFOX or CUFOX. Kaplan–Meier plots show (A, B) PFS and OS in per-protocol populations (*n* = 9 for FOLFOX, *n* = 15 for CUFOX) and (C, D) PFS and OS in those patients that did not go on to receive surgical intervention (*n* = 7 for FOLFOX, *n* = 16 for CUFOX). Survival time is shown in days. OS and PFS were compared between treatment arms using log-rank tests. CUFOX, folinic acid/5-fluorouracil/oxaliplatin + 2 g oral curcumin/d; FOLFOX, folinic acid/5-fluorouracil/oxaliplatin; OS, overall survival; PFS, progression-free survival.

PFS and OS were calculated for the ITT population, PP participants, and for those that did not go on to receive surgery (**Figure 2**).

In the ITT population, the HR for PFS was 0.571 (95% CI: 0.24, 1.36; *P* = 0.200) and for OS was 0.339 (95% CI: 0.141, 0.815; *P* = 0.016).

In the PP group, median PFS was 171 d (range: 9–214 d) and 320 d (range: 175–405 d) for FOLFOX and CUFOX, respectively, with 6-mo survival proportions of 33.3% (95% CI: 7.8%, 62.3%) for FOLFOX and 73.3% (95% CI: 43.6%, 89.1%) for CUFOX and an HR of 0.549 (95% CI: 0.225, 1.34; *P* = 0.183). Median OS was 200 d (range: 9–563 d) and 596 d (range: 323 d–still alive) for FOLFOX and CUFOX, respectively, with a 6-mo OS proportion of 55.6% (95% CI: 20.4%, 80.5%) for FOLFOX and 93.3% (95% CI: 61.3%, 99.0%) for CUFOX. The HR was 0.271 (95% CI: 0.106, 0.697; *P* = 0.004). Objective response rate (ORR) at cycle 6 for these patients was 44.4% and 66.7% for FOLFOX and CUFOX, respectively (*P* = 0.285). At 12 cycles, ORR reached significance (*P* = 0.039) and was 11.1% and 53.3% for FOLFOX and CUFOX, respectively. No complete responses were observed. At the first scan after baseline (1–3 mo) no significant differences were observed between groups, with 22% (2 of 9) and 44% (4 of 9) of FOLFOX patients exhibiting stable disease or a partial response, respectively, compared with 28% (5 of 18) (*P* = 0.756) and 56% (10 of 18) (*P* = 0.586) for CUFOX.

When excluding patients who proceeded to surgical resection, the HR for PFS was 0.152 (95% CI: 0.046, 0.501; *P* < 0.001) and for OS was 0.146 (95% CI: 0.047, 0.459; *P* < 0.001). ORR at 6 mo for these patients was 28.6% for FOLFOX and 50.0% for CUFOX (*P* = 0.340).

### Curcuminoid analysis

Curcuminoids were not detected in baseline samples from either arm. Curcumin glucuronide is the major curcumin metabolite after oral administration, persisting in plasma for ≤24 h. One FOLFOX participant subsequently had detectable concentrations of curcumin glucuronide and curcumin sulfate, similar to concentrations previously shown to be commensurate with dietary intake via food consumption (20). In the CUFOX arm, 1 participant took capsules <80% of the time, and 2 participants had plasma curcumin glucuronide concentrations <1.00 pmol/mL (below concentrations expected from dietary intake of turmeric) and were therefore not included in the PP analyses.

In the CUFOX arm (pre–cycle 2), mean curcumin glucuronide concentrations were 32.7 pmol/mL (range: 9.17–96.0 pmol/mL), curcumin sulfate concentrations were 2.06 pmol/mL (range: 0.28–8.35 pmol/mL), curcumin concentrations were 90.8 fmol/mL (range: 0.00–251 fmol/mL), and demethoxycurcumin concentrations were 21.9 fmol/mL (range: 0.00–93.8 fmol/mL). At the end of the study, concentrations of curcumin glucuronide were below limits of detection in 13 of 15 evaluable samples, because patients had already come off trial so were no longer receiving any intervention. Example mass spectra are shown in **Supplemental Figure 2**.

### CXCL1 analysis

In explant cultures, tumor CXCL1 concentrations predicted the response to oxaliplatin + curcumin (13). Plasma analysis of CXCL1 in PP populations revealed mean baseline plasma concentrations to be 370 pg/mL (range: 94.3–891 pg/mL) and 180 pg/mL (range: 68.0–374 pg/mL) for FOLFOX and CUFOX, respectively. No significant changes over time were observed in either arm (*P* = 0.712 at trial end). CXCL1 values showed a weak negative linear relation with ORR at pre–cycle 3 (Pearson's coefficient *r* = −0.3133, *P* = 0.167).

## Discussion

This is, to our knowledge, the first randomized controlled trial of curcumin in metastatic colorectal cancer. A number of colorectal cancer studies have focused on pharmacokinetic and pharmacodynamic parameters for curcumin as a single agent, demonstrating safety and tolerability, but with limited opportunity for efficacy assessments (3–6, 23). Subsequent studies have assessed potential for efficacy in combination with chemotherapy in pancreatic (24, 25), prostate (26), and breast cancers (27), showing little additional toxicity over that observed for standard-of-care chemotherapy, and a possibility for therapeutic benefit. However, no studies as far as we know have yet compared directly to standard-of-care as part of a randomized controlled trial.

Here, we demonstrate significant OS differences between FOLFOX and CUFOX participants in the ITT, PP, and nonsurgical intervention groups. Despite these observations, we must be aware of issues which may contribute to survival bias in such small cohorts. Firstly, despite the 2 study populations being similar in demographics and Eastern Cooperative Oncology Group status, they exhibited differences in tumor staging and number of metastatic sites. Recent analyses from the Analysis and Research in Cancers of the Digestive System database stratified a variety of prognostic associations with PFS and OS in metastatic colorectal cancer (28, 29), with presence of ≥2 metastatic sites significantly associated with higher likelihood of death. In the FOLFOX cohort 77.8% participants exhibited ≥2 metastatic sites at enrolment, compared with 44.5% in the CUFOX cohort. In contrast, the CUFOX cohort contained 22.2% patients with peritoneal metastases which are also associated with significant reduction in PFS and OS (29). A further issue to be explored is that of OS for both groups. Despite the significant improvement observed in the CUFOX compared with the FOLFOX group, median OS rates in the ITT and PP CUFOX groups were in line with expectations for standard FOLFOX chemotherapy (30). Patients randomly assigned to FOLFOX had poorer OS than might be expected, which may reflect the difference in the number of metastatic sites and small patient number confounders.

The primary outcome of this trial was to assess safety and tolerability of FOLFOX chemotherapy when in combination with daily oral curcumin. The greater number of AEs observed in the CUFOX arm likely reflects the greater number of chemotherapy cycles received. Comparison of AE types between arms revealed similar trends and were in line with previously published data for FOLFOX alone (31). AEs associated with curcumin use in colorectal patients are primarily gastrointestinal, making specific attribution of causality difficult. However, 1 patient on CUFOX had gastrointestinal symptom resolution once curcumin was stopped.

A decline in quality of life via self-assessment reporting can be observed earlier than just via use of Common Toxicity Criteria-Adverse Event reporting (32) and so the Quality of Life Questionnaire-C30 remains an important tool in the concurrent assessment of safety and tolerability. Despite the low numbers, CUFOX patients had smaller negative changes to functional, symptom, and global health scores post trial than FOLFOX patients had. There is also increasing evidence that curcumin can decrease neuropathic pain and neuronal functional abnormalities in models of diabetic neuropathy, nerve injury, and cisplatin-induced neuropathy (33–35). Although this has not previously been assessed in clinical cohorts to our knowledge, we hypothesized that curcumin may similarly help to prevent oxaliplatin-induced neuropathy in CUFOX patients, with patient-reported assessments undertaken using a partially validated Gynecologic Oncology Group neurotoxicity questionnaire (36). No significant differences were reported, although slightly higher neuropathy scores were observed in FOLFOX than in CUFOX patients and so may warrant further exploration in future studies.

Analysis of plasma curcuminoid concentrations served to assess protocol compliance. Curcumin glucuronide concentrations were previously established following dosing using 500 mg oral curcumin/d in the phase I arm of this study. One-sixth of the dose (30 mg) represents a typical dietary intake (20), which would equate to plasma concentrations of ∼1.00 pmol/mL. Two patients were therefore omitted from PP analyses based on concentrations of plasma curcumin glucuronide <1.00 pmol/mL. However, it was not possible to standardize the time between taking curcumin and the patients being seen in clinic, so despite the glucuronide conjugate being known to persist for ≤36 h after very high doses (10 g) of curcumin (37), it is possible that low glucuronide concentrations in this study (using one-fifth of the 10 g dose) were caused by metabolite elimination due to prolonged postdose sampling rather than by protocol noncompliance.

There is increasing evidence that the chemokine CXCL1 is associated with metastatic spread in colorectal cancer (38), with high CXCL1 tumor expression associated with poorer prognosis and survival (39). In patient-derived explant cultures of colorectal liver metastases, high baseline CXCL1 expression was associated with response to oxaliplatin when combined with curcumin (13), likely due to the ability of curcumin to downregulate the CXCL1 transcriptional regulator, NF-κB. Although there was no significant difference in plasma CXCL1 concentrations after curcumin treatment, mean baseline concentrations were 1.7-fold higher in FOLFOX patients than in CUFOX patients. Zhuo et al. (39) showed CXCL1 tumor expression to be highly correlated with tumor diameter and M stage, leading to speculation that the higher plasma concentrations observed in FOLFOX patients may also be indicative of the similar clinicopathological characteristics and poorer OS in this particular cohort.

It is acknowledged that addition of a placebo control would have added further credence to this study; however, it is well recognized that addition of a placebo adds significantly to study cost (40). This was a major consideration in the design of this trial because the additional costs associated with manufacturing and supplying a placebo would have been prohibitive. Curcumin itself was classified as an investigational medicinal product by the UK Medicines and Healthcare products Regulatory Agency, with ensuing requirement to encapsulate and Qualified Person release increasing the original drug cost by nearly 20-fold. In addition, sourcing a placebo that was yellow in color without using E100 (curcumin) as a colorant was problematic. Although this could have nominally been overcome using opaque colored capsules, these are easy to open with potential for participants to deduce treatment assignation. These confounders ultimately precluded placebo use in the CUFOX trial.

In conclusion, here we present the first randomized controlled trial for curcumin in combination with FOLFOX chemotherapy for patients with metastatic colorectal cancer. Despite significant caveats that relate to the small study size, combination of curcumin with FOLFOX chemotherapy represents a safe and tolerable treatment with potential to provide patient benefit. To further assess curcumin as an adjunct to standard-of-care platinum-based chemotherapy, a phase III trial is now warranted.

## Supplementary Material

nxz029_Supplemental_Files

## Notes

Supported by Hope Against Cancer grants 01 (to LMH) and 02 (to ALT), The Royal College of Surgeons grant 01 (to GRBI), Bowel Disease Research Foundation grant 01 (to GRBI), Cancer Research UK grant C325/A13101 (to KB), and by Cancer Research UK with the Department of Health via Experimental Cancer Medicine Centre grants C325/A15575 and C10604/A25151 (to WPS).

Author disclosures: LMH, COOI, GRBI, SB, HW, ZS, NG-T, RS, NF, SRP, BM, WPS, AG, ALT, and KB, no conflicts of interest.

Supplemental Table 1 and Supplemental Figures 1 and 2 are available from the “Supplementary data” link in the online posting of the article and from the same link in the online table of contents at https://academic.oup.com/jn/.

Abbreviations used: AE, adverse event; CUFOX, curcumin + 5-fluorouracil + folinic acid + oxaliplatin; CXCL1, C-X-C motif chemokine ligand 1; FOLFOX, folinic acid + 5-fluorouracil + oxaliplatin; FU, fluorouracil; GRO, growth-regulated oncogene; ITT, intention to treat; ORR, objective response rate; OS, overall survival; PFS, progression-free survival; PP, per protocol.
