# Supplementary material for: Curcumin Combined with FOLFOX Chemotherapy Is Safe and Tolerable in Patients with Metastatic Colorectal Cancer in a Randomized Phase IIa Trial
Source: J Nutr. 2019 May 27;149(7):1133–9. doi: 10.1093/jn/nxz029 (PMC6602900; doi:10.1093/jn/nxz029)
Supplement: nxz029_Supplemental_Files [file nxz029_supplemental_files.zip › Supplemental figure 1 R2.pdf]

Supplementary Data

Supplemental figure 1

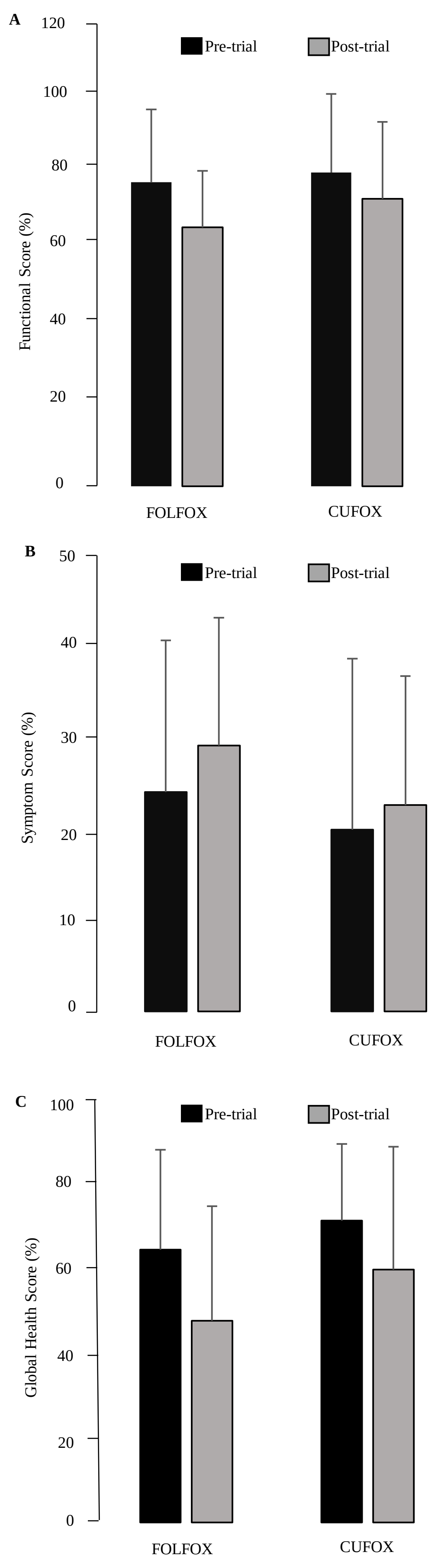

Supplemental Figure 1. Quality of life comparisons for baseline (pre-trial) participation and end of trial (post-trial) for patients with metastatic colorectal cancer receiving FOLFOX and CUFOX in the intention to treat population. (A) Functional score. (B) Symptom score. (C) Global health score. Baseline: *n* = 9 for FOLFOX; *n* = 18 for CUFOX. Follow-up: *n* = 6 for FOLFOX; *n* = 16 for CUFOX. Comparisons of EORTC-QLQ30 percentage scores between groups were made using Wilcoxon Mann-Whitney tests.
