# Supplementary material for: Curcumin Combined with FOLFOX Chemotherapy Is Safe and Tolerable in Patients with Metastatic Colorectal Cancer in a Randomized Phase IIa Trial
Source: J Nutr. 2019 May 27;149(7):1133–9. doi: 10.1093/jn/nxz029 (PMC6602900; doi:10.1093/jn/nxz029)
Supplement: nxz029_Supplemental_Files [file nxz029_supplemental_files.zip › Supplemental figure 2 R2.pdf]

# Supplementary data

**Supplemental figure 2**

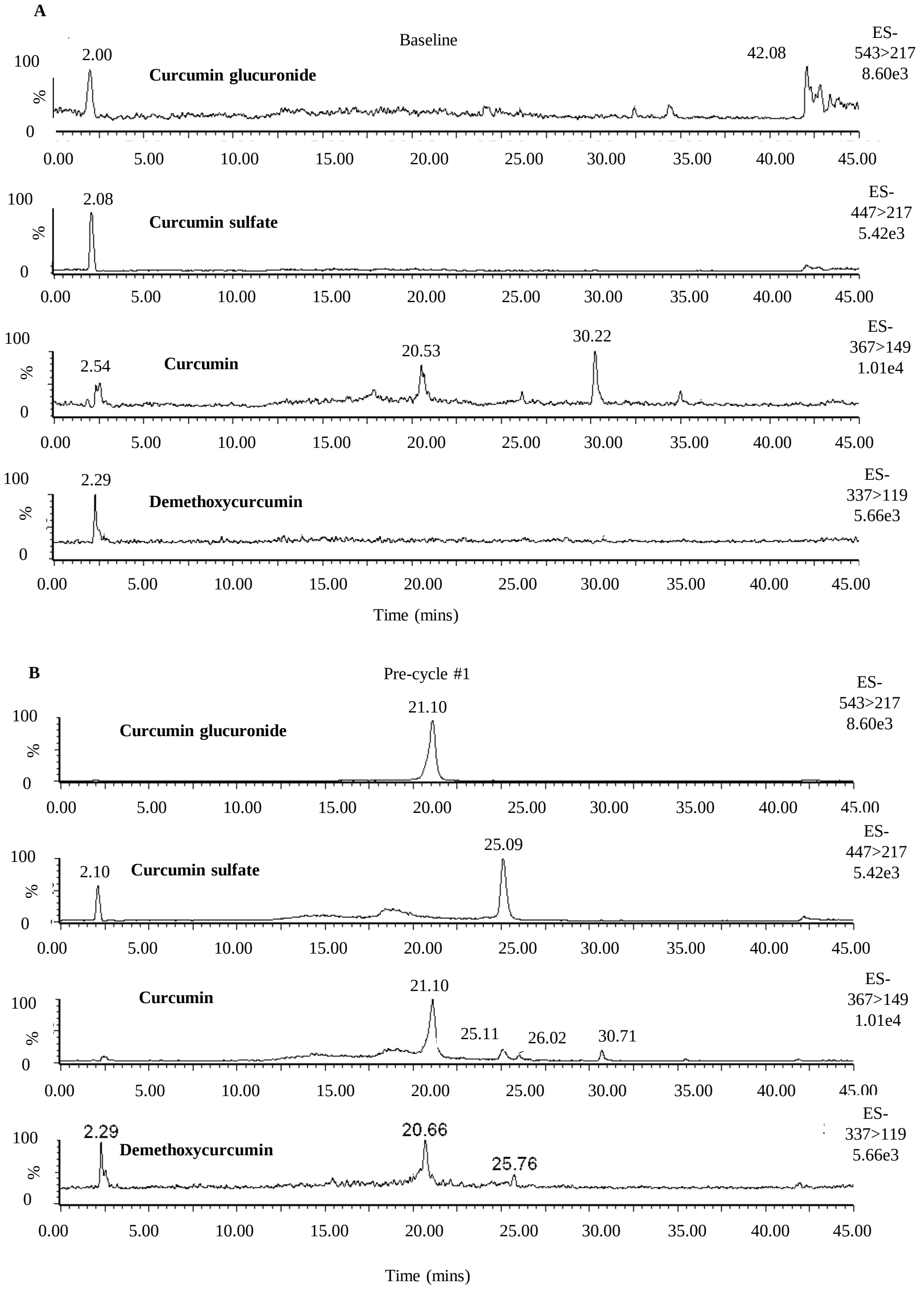


**Supplementary Figure 2**. Representative LC-ESI-MS/MS SRM chromatograms for a CUFOX patient at baseline (A) and pre-cycle 1 (B).

Samples were analyzed using LC-ESI-MS/MS with selected reaction monitoring (SRM) for the [M-H]- ion transitions of curcumin and curcumin metabolites: curcumin glucuronide 543 to 217 m/z, curcumin sulfate 447 to 217 m/z, curcumin 367 to 149 m/z and demethoxycurcumin 337 to 119 m/z. Typical LC-ESI-MS/MS SRM chromatograms are shown for plasma samples from a CUFOX patient at baseline where no curcumin or curcumin metabolite peaks were detected (A) and at pre-cycle 1 where major metabolite peaks for curcumin glucuronide and curcumin sulfate plus minor peaks for demethoxycurcumin and the parent curcumin were detected (B). The retention times were: 21.10 min for curcumin glucuronide, 25.09 min for curcumin sulfate, 26.02 min for curcumin and 25.76 min for demethoxycurcumin. The curcumin, curcumin glucuronide and curcumin sulfate concentrations were determined from authentic standard calibration lines for the different analytes. The demethoxycurcumin concentration was estimated using the curcumin calibration line, as an authentic standard was not available for this compound.
