# Supplementary material for: Curcumin Combined with FOLFOX Chemotherapy Is Safe and Tolerable in Patients with Metastatic Colorectal Cancer in a Randomized Phase IIa Trial
Source: J Nutr. 2019 May 27;149(7):1133–9. doi: 10.1093/jn/nxz029 (PMC6602900; doi:10.1093/jn/nxz029)
Supplement: nxz029_Supplemental_Files [file nxz029_supplemental_files.zip › Supplemental table 1 R2.pdf]

**Supplemental Table 1.** Adverse events by grade and arm for patients with metastatic colorectal cancer receiving FOLFOX or CUFOX treatment [1].

| | Any grade | | | Grade 1-2 | | | Grade 3-4 | | |
|---|---|---|---|---|---|---|---|---|---|
| | **FOLFOX** | **CUFOX** | **Total** | **FOLFOX** | **CUFOX** | **Total** | **FOLFOX** | **CUFOX** | **Total** |
| Abdominal pain, *n* (%) | 3 (33.3) | 5 (27.8) | 8 (29.6) | 2 (22.2) | 5 (27.8) | 7 (25.9) | 1 (11.1) | 0 (0) | 1 (3.7) |
| Anaemia, *n* (%) | 1 (11.1) | 4 (22.2) | 5 (18.5) | 1 (11.1) | 4 (22.2) | 5 (18.5) | 0 (0) | 0 (0) | 0 (0) |
| Anorexia, *n* (%) | 2 (22.2) | 6 (33.3) | 8 (29.6) | 1 (11.1) | 6 (33.3) | 7 (25.9) | 1 (11.1) | 0 (0) | 1 (3.7) |
| Constipation, *n* (%) | 4 (44.4) | 7 (38.9) | 11 (40.7) | 4 (44.4) | 7 (38.9) | 11 (40.7) | 0 (0) | 0 (0) | 0 (0) |
| Cough, *n* (%) | 1 (11.1) | 3 (16.7) | 4 (14.8) | 1 (11.1) | 3 (16.7) | 4 (14.8) | 0 (0) | 0 (0) | 0 (0) |
| Diarrhea, *n* (%) | 5 (55.6) | 9 (50.0) | 14 (51.9) | 4 (44.4) | 9 (50.0) | 13 (48.1) | 1 (11.1) | 1 (5.6) | 2 (7.4) |
| Dizziness, *n* (%) | 2 (22.2) | 3 (16.7) | 5 (18.5) | 2 (22.2) | 3 (16.7) | 5 (18.5) | 0 (0) | 0 (0) | 0 (0) |
| Dysguesia, *n* (%) | 2 (22.2) | 5 (27.8) | 7 (25.9) | 2 (22.2) | 5 (27.8) | 7 (25.9) | 0 (0) | 0 (0) | 0 (0) |
| Dyspepsia, *n* (%) | 4 (44.4) | 9 (50.0) | 13 (48.1) | 4 (44.4) | 9 (50.0) | 13 (48.1) | 0 (0) | 0 (0) | 0 (0) |
| Dyspnoea, *n* (%) | 0 (0) | 3 (16.7) | 3 (11.1) | 0 (0) | 3 (16.7) | 3 (11.1) | 0 (0) | 0 (0) | 0 (0) |
| Epistaxis, *n* (%) | 1 (11.1) | 5 (27.8) | 6 (22.2) | 1 (11.1) | 5 (27.8) | 6 (22.2) | 0 (0) | 0 (0) | 0 (0) |
| Fatigue, *n* (%) | 5 (55.6) | 15 (83.3) | 20 (74.1) | 5 (55.6) | 15 (83.3) | 20 (74.1) | 0 (0) | 1 (5.6) | 1 (3.7) |
| Flatulence, *n* (%) | 2 (22.2) | 3 (16.7) | 5 (18.5) | 2 (22.2) | 3 (16.7) | 5 (18.5) | 0 (0) | 0 (0) | 0 (0) |
| Headache, *n* (%) | 1 (11.1) | 3 (16.7) | 4 (14.8) | 1 (11.1) | 3 (16.7) | 4 (14.8) | 0 (0) | 0 (0) | 0 (0) |
| Hypercholesterolemia, *n* (%) | 0 (0) | 3 (16.7) | 3 (11.1) | 0 (0) | 3 (16.7) | 3 (11.1) | 0 (0) | 0 (0) | 0 (0) |
| Hypertension, *n* (%) | 2 (22.2) | 6 (33.3) | 8 (29.6) | 2 (22.2) | 6 (33.3) | 8 (29.6) | 0 (0) | 0 (0) | 0 (0) |
| Insomnia, *n* (%) | 0 (0) | 3 (16.7) | 3 (11.1) | 0 (0) | 3 (16.7) | 3 (11.1) | 0 (0) | 0 (0) | 0 (0) |
| Laryngitis, *n* (%) | 0 (0) | 3 (16.7) | 3 (11.1) | 0 (0) | 3 (16.7) | 3 (11.1) | 0 (0) | 0 (0) | 0 (0) |
| Low neutrophil count, *n* (%) | 1 (11.1) | 4 (22.2) | 5 (18.5) | 1 (11.1) | 2 (11.1) | 3 (11.1) | 0 (0) | 2 (11.1) | 2 (7.4) |
| Nausea, *n* (%) | 4 (44.4) | 7 (38.9) | 11 (40.7) | 4 (44.4) | 7 (38.9) | 11 (40.7) | 0 (0) | 1 (5.6) | 1 (3.7) |
| Oral mucositis, *n* (%) | 4 (44.4) | 9 (50.0) | 13 (48.1) | 4 (44.4) | 9 (50.0) | 13 (48.1) | 0 (0) | 0 (0) | 0 (0) |
| Oral thrush, *n* (%) | 2 (22.2) | 1 (5.6) | 3 (11.1) | 2 (22.2) | 1 (5.6) | 3 (11.1) | 0 (0%) | 0 (0) | 0 (0) |
| Pain, *n* (%) | 2 (22.2) | 5 (27.8) | 7 (25.9) | 1 (11.1) | 5 (27.8) | 6 (22.2) | 1 (11.1) | 0 (0) | 1 (3.7) |
| Palmar-Planter, *n* (%) | 2 (22.2) | 0 (0) | 2 (7.4) | 2 (22.2) | 0 (0) | 2 (7.4) | 0 (0) | 0 (0) | 0 (0) |
| Peripheral Oedema, *n* (%) | 2 (22.2) | 3 (16.7) | 5 (18.5) | 2 (22.2) | 3 (16.7) | 5 (18.5) | 0 (0) | 0 (0) | 0 (0) |
| Peripheral Sensory Neuropathy, *n* (%) | 4 (44.4) | 16 (88.9) | 20 (74.1) | 4 (44.4) | 16 (88.9) | 20 (74.1) | 0 (0) | 2 (11.1) | 2 (7.4) |
| Platelet count decreased, *n* (%) | 2 (22.2) | 8 (44.4) | 10 (37.0) | 2 (22.2) | 8 (44.4) | 10 (37.0) | 0 (0) | 1 (5.6) | 1 (3.7) |
| Pyrexia, *n* (%) | 2 (22.2) | 4 (22.2) | 6 (22.2) | 1 (11.1) | 4 (22.2) | 5 (18.5) | 1 (11.1) | 0 (0) | 1 (3.7) |
| Raised Alkaline Phosphatase, *n* (%) | 0 (0) | 3 (16.7) | 3 (11.1) | 0 (0) | 2 (11.1) | 2 (7.4) | 0 (0) | 1 (5.6) | 1 (3.7) |
| Rash, *n* (%) | 1 (11.1) | 3 (16.7) | 4 (14.8) | 1 (11.1) | 3 (16.7) | 4 (14.8) | 0 (0) | 0 (0) | 0 (0) |
| Respiratory tract infection, *n* (%) | 0 (0) | 3 (16.7) | 3 (11.1) | 0 (0) | 3 (16.7) | 3 (11.1) | 0 (0) | 0 (0) | 0 (0) |
| Sepsis, *n* (%) | 2 (22.2) | 0 (0) | 2 (7.4) | 0 (0) | 0 (0) | 0 (0) | 2 (22.2) | 0 (0) | 2 (7.4) |
| Skin infection, *n* (%) | 0 (0) | 3 (16.7) | 3 (11.1) | 0 (0) | 2 (11.1) | 2 (7.4) | 0 (0) | 1 (5.6%) | 1 (3.7) |
| Thromboembolic event, *n* (%) | 0 (0) | 4 (22.2) | 4 (14.8) | 0 (0) | 1 (5.6) | 1 (3.7) | 0 (0) | 3 (16.7) | 3 (11.1) |
| Urinary tract infection, *n* (%) | 1 (11.1) | 3 (16.7) | 4 (14.8) | 1 (11.1) | 3 (16.7) | 4 (14.8) | 0 (0) | 0 (0) | 0 (0) |
| Vomiting, *n* (%) | 0 (0) | 6 (33.3) | 6 (22.2) | 0 (0) | 6 (33.3) | 6 (22.2) | 0 (0) | 1 (5.6) | 1 (3.7) |
| Weight Loss, *n* (%) | 2 (22.2) | 3 (16.7) | 5 (18.5) | 2 (22.2) | 2 (11.1) | 4 (14.8) | 0 (0) | 1 (5.6) | 1 (3.7) |

[1]Values represent frequency (percent) of adverse events by grade and arm for intention to treat population, reported for those events that constituted ≥15% of all adverse events. FOLFOX, folinic acid/5-fluorouracil/oxaliplatin; CUFOX, folinic acid/5-fluorouracil/oxaliplatin + 2 g daily oral curcumin. Number of patients on FOLFOX and CUFOX in intention to treat population was 9, and 18, respectively.
